# LEARNING YOGA ONLINE: PERSPECTIVES FROM OLDER WOMEN ENROLLED IN A CLINICAL TRIAL FOR URINARY INCONTINENCE

**DOI:** 10.1093/geroni/igae098.0241

**Published:** 2024-12-31

**Authors:** Francesca Nicosia, Margaret Chesney, Celia Kaplan, Berty Arreguin, Giselle Perez-Aguilar, Alison Huang

**Affiliations:** University of California San Francisco, San Francisco, California, United States; University of California San Francisco, San Francisco, California, United States; University of California San Francisco, San Francisco, California, United States; University of California San Francisco, San Francisco, California, United States; University of California San Francisco, San Francisco, California, United States; University of California San Francisco, San Francisco, California, United States

## Abstract

Yoga is increasingly used as a therapeutic modality for aging-related health conditions but the feasibility of engaging older adults in yoga is unclear. We conducted a multisite randomized trial of a therapeutic yoga intervention for ambulatory older women with urinary incontinence. The trial included twice weekly group instruction in Hatha yoga techniques delivered via interactive, synchronous, videoconference-based instruction. To assess barriers and facilitators to online instruction for this population, we used mixed methods. Structured questionnaires administered at 12-weeks measured convenience, audio-visual elements, and social connection, each on a 4-pt scale (1: Poor, 4: Excellent). Interviews examined the experience of learning yoga through synchronous video instruction at home. A hybrid deductive/inductive thematic approach was used to analyze interview transcripts. Among the 51 participants completing questionnaires (age range 49-81; 16% Asian, 16% Latina, 71% White), ratings of convenience were high (90.3% “good-excellent”); ratings of perceptions of personal connection with other participants were lower (60.8% “good-excellent”). Among the 24 participant interviews (age range 45-90; 21% Asian, 17% multiracial; 17% Latina, 54% White), common challenges were audio-visual and physical space limitations. For some, privacy of the home setting mitigated incontinence stigma. Increased body awareness and perceived effectiveness in reducing incontinence and stress facilitated sustained participation. Participants identified effective online teaching strategies including reinforcement of verbal instructions with visual demonstrations about body position during yoga postures and individualized attention. Despite challenges with online delivery, the convenience and privacy of the at-home setting provides opportunities to engage diverse, older women in movement-based therapeutic yoga interventions.

